# Comparison of 2 Fillers for Lip Injection—A Randomized-Controlled Clinical Trial Assessed by 3D Imaging

**DOI:** 10.1093/asjof/ojae003

**Published:** 2024-01-18

**Authors:** Marcelo Germani, Claudia Cristina Miranda de Souza Almeida, Victor Ricardo Manuel Muñoz-Lora

## Abstract

**Background:**

Lip aging is a concern for many, and hyaluronic acid (HA) injections are a popular solution.

**Objectives:**

This study compared 2 different HA gel technologies (OBT and NASHA) for lip augmentation in 20 volunteers.

**Methods:**

Both groups received treatment from the same injector using the same method. Lip volume was measured with a 3D stereophotogrammetry device before, immediately after, and 30 days posttreatment. Patient satisfaction and adverse events were assessed through FACE-Q scales analysis.

**Results:**

The NASHA group showed an immediate volume increase (*P* = .01), which decreased after 30 days. The OBT group did not show a significant immediate growth (*P* = .535) but did exhibit a significant increase after 30 days (*P* = .014). After 30 days, there were no significant volume differences between the groups (*P* = .802 and *P* = .999). FACE-Q analysis revealed no significant differences among groups after 30 days.

**Conclusions:**

This study highlights that less cohesive gels may modify faster in dynamic lip areas, emphasizing the importance of selecting products based on their physicochemical and rheological properties. In the context of lip augmentation with HA, it is important not to consider immediate aesthetic changes as definitive results, as volume changes may persist and develop over time after the treatment.

**Level of Evidence: 2:**

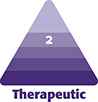

Lips play an essential functional, aesthetic, and emotional role. Young and attractive lips assume striking anatomical characteristics, which are clearly altered during the aging process.^1,2^ Lip filling with hyaluronic acid (HA) is a minimally invasive procedure that seeks to enhance lips or alleviate lip aging. However, restructuring this region must be carefully evaluated due to the high complexity of movements and expressions performed by the lips.^3-5^

The appropriate HA for lip filling should combine specific characteristics such as high cohesiveness, good flexibility, and small or medium particle size,^3,6,7^ to deal with the high mobility of this region. Nevertheless, the market offers a diverse range of HA gels with varying physicochemical and rheological properties, adding complexity to the decision-making process for selecting the most suitable product.^8^

Current assessments for lip enhancement are usually based on subjective questionnaires and analysis. Literature lacks comparative studies using well-defined patterns, such as 3D volumetric analyses, which allow for a more objective assessment of the results.^9,10^ On the contrary, quantitative and qualitative standards regarding the satisfaction of individuals are extremely relevant to identify which product performs better in the region.^3^

For these reasons, the aim of the present study was to compare the volume augmentation of lips after injection of 2 fillers with different rheological properties, as well as patient satisfaction and the incidence of adverse events among them.

## METHODS

### Volunteers

This study was approved by the Clinical Research Ethics Committee of the Santo Amaro University (UNISA, CAAE no. 65762322.3.0000.0081—Date: February 20, 2023) and followed the Declaration of Helsinki guidelines. All patients provided written informed consent prior to inclusion. The study adhered to the Consort Statement guidelines. It was registered at REBEC, the clinical trial platform of Brazil (UTN: U1111-1302-7984).

**Figure 1.**
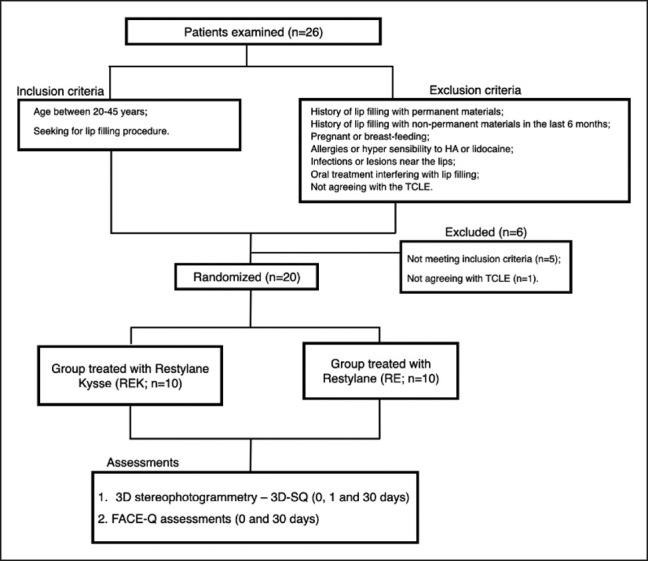
Flow diagram.

Twenty volunteers between 20 and 45 years old seeking lip filler treatment were recruited from February to March 2023 (Table). Patients with a history of lip filling with permanent or nonpermanent materials within a period of less than 6 months were excluded from the study. Pregnant or breastfeeding females and patients with allergies or hypersensitivity to any HA gel, lidocaine, or other amide-type anesthetics were also excluded. Patients with infections or lesions in or near the area to be treated, oral or dental treatments that may interfere with injections or study evaluations, and patients who did not agree to sign an informed consent form were excluded (Figure 1).

**Table.** Demographics

	
Volunteers	20
Male	1
Female	19
Mean age	32 ± 12 years

	Initial	Immediately after (mm)	30 days (mm)
Mean lip volume	17.73	19.79	19.59
Bigger lip	23.54	25.24	24.01
Smaller lip	14.31	15.54	15.8

### Procedure

Volunteers were randomly divided into 2 groups (*n* = 10) using random allocation software (GraphPad, Dotmatics):

1. *Group RE*—Treated with 1 mL of Restylane (Galderma, Upsalla, Sweden), featuring nonanimal stabilized HA (NASHA) technology.
2. *REK Group***—**Treated with 1 mL Restylane Kysse (Galderma), with Optimal Balance (OBT)/XpresHAn technology.

Patients were prepared in advance by thoroughly washing their faces with water and neutral soap, followed by asepsis using 2% aqueous chlorhexidine and a mouthwash with 0.2% chlorhexidine. All patients received treatment from the same injector using the exact same injection technique described below.

Anesthesia was administered at the labial commissure with lidocaine (DFL, São Paulo, Brazil) without a vasoconstrictor, using a 32 G hypodermic needle (Terumo, São Paulo, Brazil). For lip filling, a cannula entry point was established exactly 1 cm away from both labial commissures using a 24 G needle (Terumo, São Paulo, Brazil). Subsequently, a 22 G cannula (Terumo, São Paulo, Brazil) was used for linear retroinjection of 0.25 mL (1/4 of the total product) bilaterally in the submucosal plane, both in the upper and lower lip. No manipulation or massage was performed after the procedure (Video).

All patients received instructions regarding the postoperative period and were advised not to consume hot or hard foods for a duration of 6 h, in addition to refraining from engaging in physical activity for 2 days.

### Outcomes

All outcomes were evaluated by the same investigator, who was unaware of the treatment.

#### 3D Stereophotogrammetry—3D-SQ (0, 1, and 30 Days)

**Figure 2.**
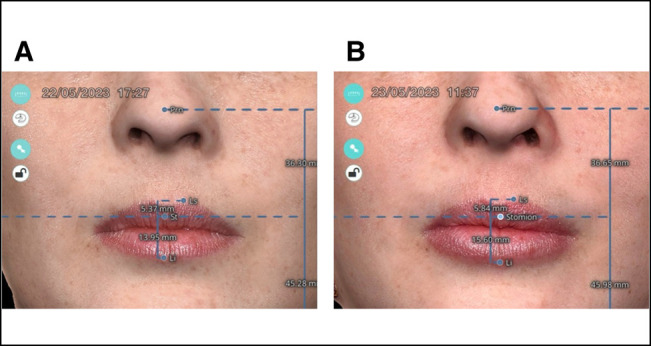
Assessment of (A) before and (B) 30 days after the procedure of a 42-year-old female volunteer, using 3D imaging with a 3D stereophotogrammetry program (Quantificare). ; Li, inferior lip point; Ls, superior lip point; Prn, pronasal point; St, stomion point.

The volume assessment (cc) using 3D stereophotogrammetry (3D-SQ) was performed with a device (Quantificare, Sophia Antipolis, France) capable of capturing 2 images simultaneously from different angles. After obtaining these images, specialized software (DermaPix Database for photographic documentation, Quantificare) was employed to process and create a 3D reconstruction. The LifeViz application, designed for computing and managing 3D images, calculated and highlighted the differences between the analyzed images. All photographs were taken by the same blinded operator and adhered to strict patient positioning in a standardized manner (Figure 2).

#### FACE-Q Assessments (0 and 30 Days)

The FACE-Q encompasses a collection of independent scales and checklists designed to assess crucial concepts and symptoms for facial aesthetic patients across various facial areas. The FACE-Q scales can be administered to any facial aesthetic patient, whether they have undergone surgical or nonsurgical procedures, to gauge patients' perceptions of their appearance, quality of life, adverse effects, and the care process.^11^

Two domains of the FACE-Q questionnaire were administered: outcomes and lip satisfaction. The questionnaires were conducted through interviews conducted by the same researcher. The time required to complete the 2 domains was approximately 15 min. The FACE-Q questionaries were conducted using Google form surveys sent to the patients a day before the appointment.

### Statistical Analysis

Quantitative data were presented as mean ± standard deviation (SD). To assess significant differences (*P* < .05) among groups over time, the data were analyzed using 2-way repeated measures analysis of variance (RM ANOVA), followed by Tukey’s post hoc test for intertreatment comparisons.

The percentage of volume changes (PVCs) over time was calculated, and independent sample *t* tests were employed to compare these changes among groups.

Responses from the FACE-Q outcomes and FACE-Q lips questionnaires were compiled in a Microsoft Excel sheet. For each FACE-Q section (outcomes and lips), the responses were aggregated and then transformed into a 0 (lowest satisfaction) to 100 (highest satisfaction) point scale using Rasch measurement methods. Subsequently, RM ANOVA was applied to identify any significant changes between the treated groups (Restylane Kysse vs Restylane) and across time points (before vs after).

All statistical analyses were conducted using Jamovi (The Jamovi Project, version 1.6.23).

## RESULTS

The sample comprised 19 female and 1 male volunteers with an average age of 32 (±12) years. All patients were assessed for 30 days after treatment. The mean initial lip volume for the RE group was 17.74 mm ± 2.78, and for the REK group, it was 17.92 mm ± 2.67. No serious adverse events were reported by patients.

### 3D Stereophotogrammetry

**Figure 3.**
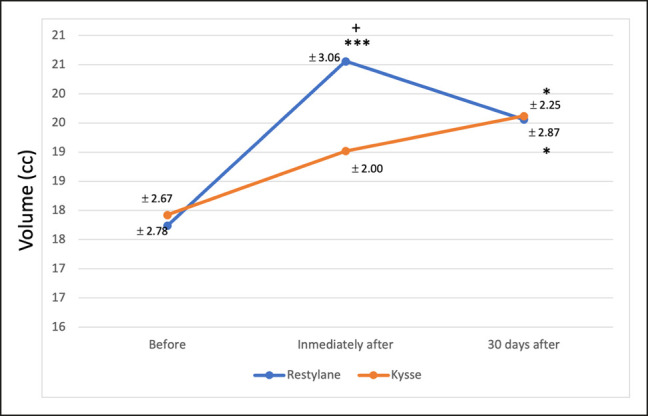
Volume increase in lips over time. ***P < .001 vs baseline (RM ANOVA, Tukey test); *P < .05 vs baseline (RM ANOVA, Tukey test); +*P* < .05 among groups (RM ANOVA, Tukey test).

Intragroup analysis revealed a significant increase in lip volume immediately after treatment in the RE group (*P* = .001; from 17.74 mm ± 2.78 to 20.56 mm ± 3.06). However, this initial increase decreased after 30 days to 19.56 ± 2.87 mm. The intragroup analysis for REK showed a nonsignificant volume increase (*P* = .535; from 17.92 mm ± 2.67 to 19.02 mm ± 2.00) immediately after treatment. After 30 days, a significant increase in lip volume was observed when compared to the initial values (*P* = .014; from 17.92 mm ± 2.67 to 19.62 mm ± 2.25; Figure 3).

**Figure 4.**
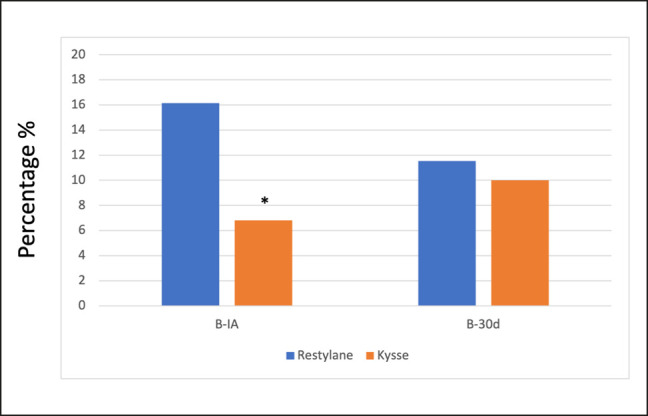
Percentage of volume change. **P < .01 (independent *T* test). B, baseline; IA, immediately after; 30d, 30 days.

When comparing the results obtained 1 and 30 days after treatment among groups, no significant differences in volume were found (*P* = .802 and *P* = .999, respectively). Finally, the PVC over time was also calculated. PVC is defined as the percentage of volume increase/decrease from baseline to any timepoint. Immediately after treatment, RE volunteers experienced a significantly higher baseline volume than REK volunteers (16.14% ± 5.68 vs 6.81% ± 7.46; *P* = .006). When comparing the PVC at baseline and 30 days, no significant differences were found between groups (11.53% ± 10.8 for RE and 9.98% ± 5.39 for REK; *P* = .697; Figure 4).

### FACE-Q Analysis

**Figure 5.**
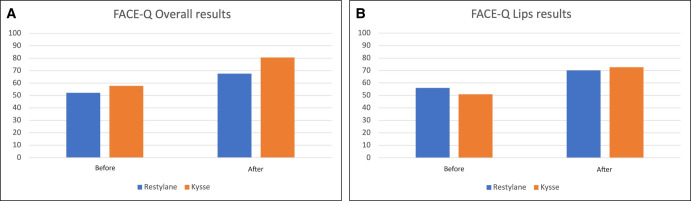
FACE-Q (A) overall and (B) lips results.

The FACE-Q analyses indicate that before and 30 days after treatment, the domain satisfaction with the result and satisfaction with the lips were similar between the RE and REK groups (*P* = .469 and *P* = .624, respectively; Figure 5).

## DISCUSSION

When proposing lip treatments with HA, it is essential to consider the physicochemical characteristics and rheology (ie, the behavior of the gel when subjected to deforming forces) of the chosen gel.^3,7^ The correct properties of the products will result in more efficient volumization, meaning better results with less product and more natural outcomes. Additionally, qualitative and quantitative standards related to patient satisfaction and perception are extremely relevant, particularly in areas with significant and constant mobility, such as the lips.^3,8^

In this study, patients were treated with 2 HA fillers indicated for volumization and/or rejuvenation of the lips and perioral region. Our results demonstrated a similar behavior between the products after 30 days (*P* > .05). Interestingly, a progressive volumetric increase (measured by 3D-SQ) was observed immediately and after 30 days with Restylane Kysse. In contrast, Restylane showed a significant initial increase followed by a decrease in the subsequent evaluation (30 days).

RE exhibited greater lip projection than REK immediately after treatment. This difference can be attributed to the specific physicochemical and rheological characteristics of the products. The production technology of each product plays a critical role in its behavior. Restylane is produced using NASHA technology, characterized by natural bonds and stabilization of HA with homogeneous particles that incorporate only 1% of crosslinking. Restylane has a G prime of 544 Pa, making it a firm gel, ideal for projection and accuracy.^12^ On the contrary, Restylane Kysse is a more flexible filler due to the OBT technology. It is a small-particle gel with 156 Pa of G prime, providing greater integration in dynamic regions.^13,14^ These rheological properties might account for the significant immediate volume increase observed in the RE group when compared with the baseline, whereas the REK group showed a nonsignificant increase in comparison to the baseline.

PVC results corroborate this initial significant increase in RE. However, this difference is no longer found after 30 days (*P* > .05). This result can be explained by the different cohesiveness of the products. Cohesiveness can be defined as the bond strength between HA particles, and it is a relevant feature for maintaining the crosslink and the structural shape of the gel.^15^ Several authors have emphasized the importance of cohesiveness in dynamic areas.^16,17^

It was demonstrated that firmer products with a high projection capacity can be more easily degraded in high mobility areas, such as the lips.^1^ Restylane has a high projection capacity due to its minimal lateral flow and high G prime. However, this initial projection is not sustained over time due to its low cohesivity and less tissue integration in a high mobility area.

Another important consideration is the different swelling factors between the products. Due to its more flexible characteristics, Restylane Kysse can easily alter its volume and has a higher hydrophilic capacity with a progressive volume increase over time.^13^ In contrast, Restylane has a lower swelling factor due to its higher G prime, making it less prone to absorbing water from the surrounding tissue and promoting a very subtle volume increase.^18^

As a strength of our study, we can highlight the 3D analysis, which can be considered an objective assessment for volumetric changes. However, despite their high reliability, of 3D analysis can present a coefficient variation of 2.8% to 6.8%, which can represent a limitation of our study.^19^ Another limitation may be the sample size, despite our assessments being highly reliable and objective.

A longer follow-up time may also help further elucidate and diminish the differences between both products when applied to the lips.

## CONCLUSIONS

The use of 3D-SQ provides an objective assessment of lip volumetric changes following filler injection. Although there were no significant volumetric differences among the groups at the endpoint, our findings shed light on the distinct behaviors exhibited by these products. Specifically, RE exhibited a substantial initial volumetric increase that was partially diminished after 30 days, whereas REK demonstrated a progressive increase over time.

This study highlights that less cohesive gels may modify faster in dynamic lip areas, emphasizing the importance of selecting products based on their physicochemical and rheological properties. In the context of lip augmentation with HA, it is important not to consider immediate aesthetic changes as definitive results, as volume changes may persist and develop over time after the treatment.
